# Supplementary material for: Dissipative shock waves generated by a quantum-mechanical piston
Source: Nat Commun. 2018 Nov 7;9:4665. doi: 10.1038/s41467-018-07147-4 (PMC6220177; doi:10.1038/s41467-018-07147-4)
Supplement: Supplementary file 1 — Supplementary Information [file 41467_2018_7147_MOESM1_ESM.pdf]

Dissipative shock waves generated by a quantum-mechanical piston

Mossman *et al*

SUPPLEMENTAL MATERIAL

Supplementary Note 1

The Gross-Pitaevskii equation

$$i\psi_t = -\frac{1}{2}\nabla^2\psi + V\psi + |\psi|^2\psi, \quad \mathbf{r} \in \mathbb{R}^3, \quad t > 0 \tag{1}$$

is used to model the quantum piston problem whereby a BEC is condensed to its ground state in a cigar-shaped harmonic trap

$$V_{\text{ho}}(x, y, z) = \frac{1}{2}((\lambda_x x)^2 + y^2 + (\lambda_z z)^2), \tag{2}$$

$$\lambda_x = 0.969 \approx 1, \quad \lambda_z = 0.01 \ll 1. \tag{3}$$

The long axis of the BEC is along the z-axis. The piston is a repulsive laser sheet that is swept across the trapped BEC, modeled by a moving Gaussian potential

$$V_{\text{p}}(x, y, z) = V_0 \exp\left[-\frac{x^2 + y^2}{2s_\rho^2} - \frac{z^2}{2s_z^2}\right], \tag{4}$$

$$V_0 = 155, \quad s_\rho = 95.3, \quad s_z = 15.4. \tag{5}$$

The potential in eq. (1) consists of the piston translated axially and the stationary trap

$$V(x, y, z, t) = V_{\text{ho}}(x, y, z) + V_{\text{p}}(x, y, z + v_{\text{p}}t - z_0), \tag{6}$$

with piston velocity v_{p} and the initial piston location $z_0 = 365$.

These are dimensionless equations. The corresponding dimensional spatial, temporal, and density scales are $a_y = \sqrt{\hbar/(m\omega_y)}$, $T = 1/\omega_y$, and $\Gamma = 1/\sqrt{4\pi a_{\text{s}} a_y^2}$, respectively.

Equation (1) can be written in hydrodynamic form with the exact transformation $\psi = \sqrt{n}e^{i\phi}$, $\mathbf{u} = \nabla\phi$

$$\begin{aligned} n_t + \nabla\cdot(n\mathbf{u}) &= 0 \\ (n\mathbf{u})_t + \nabla\cdot(n\mathbf{u}\otimes\mathbf{u}) + \frac{1}{2}\nabla n^2 &= \frac{1}{4}\nabla(n\nabla\otimes\nabla\log n) - \nabla V. \end{aligned} \tag{7}$$

Restricting to one-dimensional, planar shocks $u = \mathbf{u}\cdot\hat{\mathbf{z}}$ ($\hat{\mathbf{z}} = (0, 0, 1)^T$), neglecting the potential V and the dispersive term $[n(\log n)_{zz}]_z$ yields the long-wave hydrodynamic equations

$$n_t + (nu)_z = 0 \tag{8}$$

$$(nu)_t + (nu^2 + \frac{1}{2}n^2)_z = 0. \tag{9}$$

These equations are equivalent to the shallow water equations [1]. We now solve the viscous shock problem by invoking the Rankine-Hugoniot jump relation [1]

$$-s[n] + [nu] = 0 \tag{10}$$

$$-s[nu] + [nu^2 + \frac{1}{2}n^2] = 0, \tag{11}$$

where $[\cdot]$ represents a jump in the quantity across the discontinuous shock front and s is the shock speed. If we normalize to quiescent downstream conditions $n_+ = 1$, $u_+ = 0$, then we can solve Eqs. (10) and (11) for the jump in density and velocity $n_- > n_+$, $u_- > u_+$ to give a shock satisfying

$$u_- = \frac{n_- - 1}{\sqrt{2}}\sqrt{1/n_- + 1}, \quad s = \frac{n_- u_-}{n_- - 1}. \tag{12}$$

The piston problem is solved by equating the flow speed to the piston speed $u_- = v_p$. The equations in (12) are used to obtain the theoretical viscous shock speed s and plateau density n_- curves in Fig. 2 of the main manuscript where the speed is normalized by the effective speed of sound $c_{\text{s,eff}} = 1.35$ mm s^{-1} and the density is normalized by the maximum density at the BEC center.

We note that viscous effects lead to the imposition of the jump conditions (10), (11) for the viscous shock. However, the magnitude of the viscosity does not influence these conditions. The primary role of the viscosity is to determine the shock structure, i.e., the width over which the flow transitions from (n_-, u_-) to (n_+, u_+).

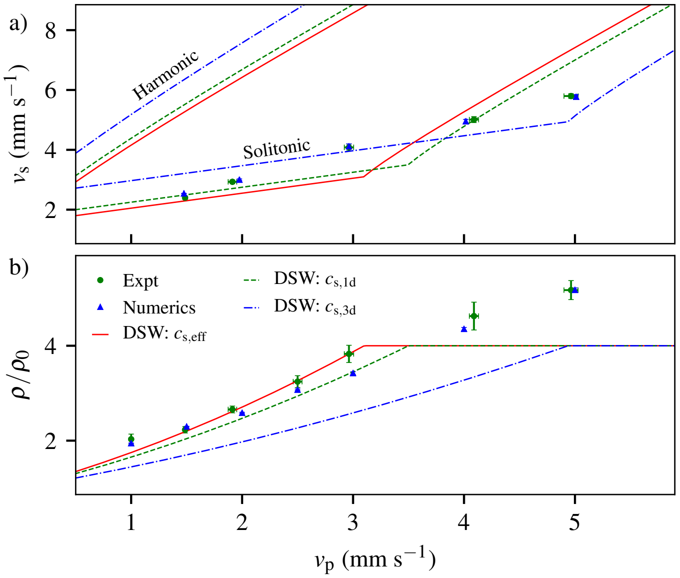

Supplementary Figure 1: *Shock Speed, Peak Height vs. Piston Speed.* Best fit of experimental (green dots) and numerical (blue triangles) data to the piston DSW closure relations with $c_{s,eff} = 1.55$ mm/s, $c_{s,1d} = 1.75$ mm/s, and $c_{s,3d} = 2.47$ mm/s. **a,** Both the DSW harmonic edge speed, v_{har} (upper curves), and soliton edge speed, v_{soli} (lower curves), are plotted. For the detailed definition of these quantities, see Supplementary Note 2. **b,** The maximum normalized peak height with respect to DSW theory. For DSW theory, the saturation of the plateau height and a discontinuity of v_{soli} occur when $v_p \geq 2c_s$. Weighted mean $\pm$ s.d. are plotted for three (a) and five (b) data runs.

Supplementary Note 2

For completeness, we quote the formulas in normalized units of the sound speed c_s and density for a dispersive shock wave (DSW) solution, i.e., a shock wave in which viscosity is negligible relative to dispersion [2]. In contrast to viscous shock waves, dispersive shock waves exhibit two speeds of propagation, the trailing, large amplitude soliton edge and the leading, small amplitude harmonic edge. For a piston moving with speed v_p into a quiescent BEC with unit density and zero velocity, the plateau density $n_- = (v_p/2+1)^2$ saturates when $v_p = 2$, twice the speed of sound in the quiescent BEC. For piston speeds larger than $v_p = 2$, the dispersive shock wave is oscillatory up to the piston and the peak normalized density oscillation is 4. The dispersive shock wave soliton edge moves with speed

$$v_{soli} = \begin{cases} v_p/2+1 & 0 < v_p \leq 2, \\ v_p - 2\left[1 - \frac{v_p E(4/v_p^2)}{(v_p-2)K(4/v_p^2)}\right]^{-1} & v_p > 2, \end{cases}$$

where K and E are the complete elliptic integrals of the first and second kind. Note that there is an error in [2] for the dispersive shock wave large amplitude edge speed when $v_p > 2$. The dispersive shock wave harmonic edge moves with speed $v_{har} = (2v_p^2 + 4v_p + 1)/(v_p + 1)$ for all piston speeds $v_p > 0$. The best fit of the experimental and numerical simulation data is shown in Supplementary Fig. 1 using the speed of sound as the sole fitting parameter (as in the viscous shock case reported in Fig. 2 of the main manuscript). Here, the best fit effective speed of sound is $c_{s,eff} = 1.55$ mm/s, but the data does not agree with both the shock speed and plateau density simultaneously across the range of piston speeds surveyed.

We have verified the predictions of DSW theory by 3D numerical simulations with sufficiently tight confinement ($\lambda_x = 0.969$, $\lambda_z = 0.0010$, number of atoms $N = 8732$). In this case, the dimensionality parameter $d = 0.068$ is much smaller than unity. Additionally,

$$\left(\frac{Na_s}{\sqrt{\lambda_z}a_y}\right)^{1/3} = 12.6,$$

which is much larger than unity and therefore the ground state can be approximately factored into the Thomas-Fermi (dispersionless) approximation for the long (z) direction and the harmonic oscillator ground state for the transverse

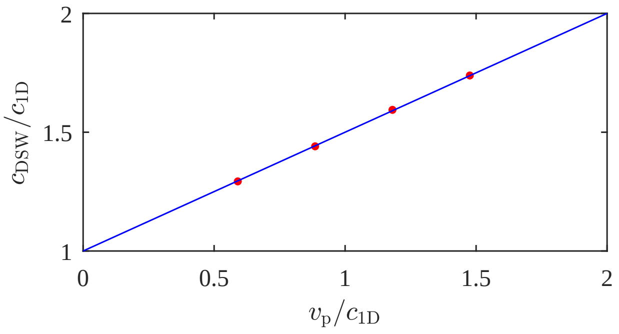

Supplementary Figure 2: *DSW trailing edge soliton speed vs. piston speed.* Comparison between 1D theory (circles) and 3D numerics (solid line).

(x-y) directions [3, 4]. This enables the approximate solution of the 3D BEC piston problem using the 1D DSW results. Supplementary Fig. 2 shows a convincing comparison between the DSW soliton edge speed $c_{\rm DSW}$ from 3D numerical simulations and the 1D theory for a range of piston speeds. The effective speed of sound is the usual 1D speed of sound $\sqrt{n_0/2}$ where n_0 is the peak density at the trap center, i.e., no fitting was used in Supplementary Fig. 2.

Supplementary Note 3

Supplementary Fig. 3a and b reports 2D incompressible and compressible kinetic energy spectra, respectively, extracted from three regions (red, blue and green) in the flow indicated in Supplementary Fig. 3c. The two rightmost intervals (blue and green) exhibit similar spectral features and a fitted $k^{-3.4}$ scaling. The reported energy spectra are the z-averaged spectra computed from spatial slices of the density-scaled velocity field $\sqrt{\rho}\mathbf{u} = \mathbf{v}_{\rm i} + \mathbf{v}_{\rm c}$, decomposed into its incompressible $\mathbf{v}_{\rm i}$ (divergence free) and compressible $\mathbf{v}_{\rm c}$ (curl free) components

$$E_{\rm i,c}(k) = \int_a^b \int_0^{2\pi} |\hat{\mathbf{v}}_{\rm i,c}(k\cos\varphi, k\sin\varphi, z)|^2 \, d\varphi dz, \tag{13}$$

where

$$\hat{\mathbf{v}}_{\rm i,c}(k_x, k_y, z) = \int_{\mathbb{R}^2} \mathbf{v}_{\rm i,c}(x, y, z) e^{-i(k_x x + k_y y)} \, dxdy \tag{14}$$

is the two-dimensional Fourier transformation in the transverse x-y directions.

Supplementary Fig. 4 reports the temporal development of the total quantum pressure energy $E_{\rm qp}(t) = \frac{1}{2}\int |\nabla\sqrt{n}|^2 \, d\mathbf{r}$, total incompressible $E_{\rm i} = \frac{1}{2}\int |\mathbf{v}_{\rm i}|^2 \, d\mathbf{r}$ and compressible $E_{\rm c} = \frac{1}{2}\int |\mathbf{v}_{\rm c}|^2 \, d\mathbf{r}$ kinetic energies where $\mathbf{v}_{\rm i}$ and $\mathbf{v}_{\rm c}$ are the divergence free and curl free components, respectively, of the scaled velocity field $\mathbf{v} = \sqrt{n}\mathbf{u}$. The quantum pressure energy highlights regions of the flow exhibiting large density gradients such as those that occur in solitons and vortex lines. Consequently, it scales with the vortex line length and therefore represents a measure of the amount of quantized vorticity. In addition to the main text's reported vortex structures, the monotonic increase of $E_{\rm qp}$ as a function of time is an additional indication of continued turbulence production as the piston is swept through the BEC.

Supplementary Note 4

To experimentally study rarefaction waves, we adiabatically sweep the barrier from the right (outside of the BEC) to the center of the BEC such that no excitations are formed during the sweep. After this sweep, all atoms are confined in the left half of the trap. The background density in the area to the right of the BEC is determined to be negligible. The barrier is then jumped off and images are subsequently taken at $t = \{10, 15, 20, 25, 30, 35, 40\}$ ms. A direct comparison to numerics can be found in Supplementary Fig. 5.

To analyze the data and corresponding numerics found in the main text, we first make an integrated cross section of the data as seen in Supplementary Fig. 5, and take the square root of this cross section. The right half of the cross

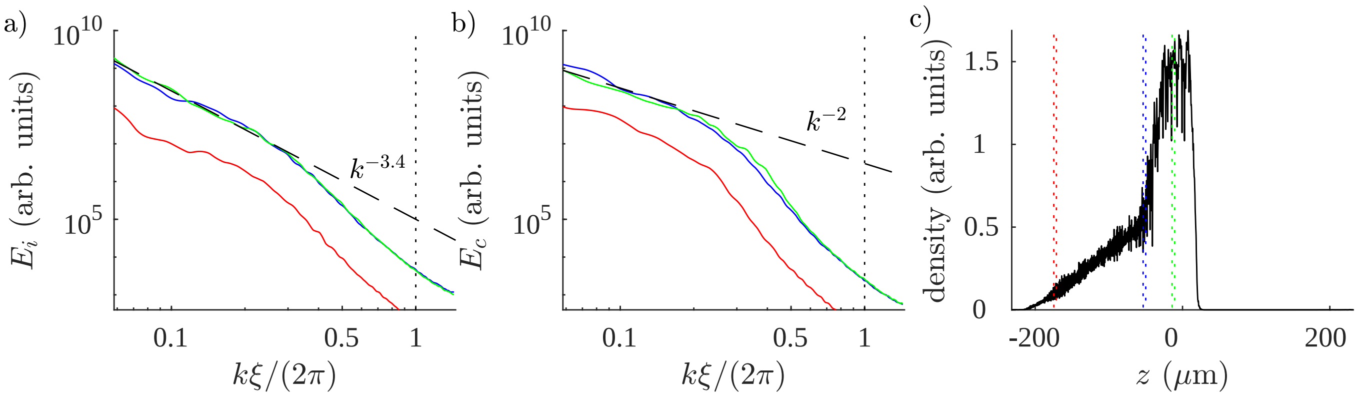

Supplementary Figure 3: *Incompressible and compressible kinetic energy spectra.* **a,** Azimuthally averaged, 2D incompressible and **b,** compressible kinetic energy spectra obtained from the average of multiple spatial slices taken transverse to the piston direction at the locations identified by correspondingly colored vertical dashed lines in **c**. The spectrum is normalized to the healing length at the trap center for the equilibrium condensate (0.21 μm) taken prior to piston motion. The incompressible and compressible spectrum exhibit power law decay $\sim k^{-3.4}$ and $\sim k^{-2}$, respectively over a range of wavenumbers.

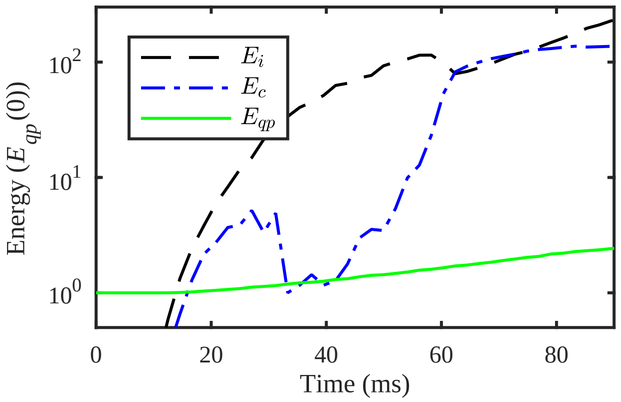

Supplementary Figure 4: *Evolution of Energies.* Total quantum pressure (green solid), incompressible (black dashed) and compressible (blue dot-dashed) kinetic energies from simulation in Fig. 3b and Fig. 4 of the main manuscript. The decrease in compressible kinetic energy at $t \approx 30$ ms coincides with the transverse (snake) instability and subsequent break-up of the transient DSW. From ≈ 40 ms onwards, the total quantum pressure, incompressible, and compressible energies increase due to the continual production of quantized vorticity and dispersive waves. All energies are normalized by the initial quantum pressure energy.

section is now linear, as expected from a quadratic profile, and is fitted with a linear trend line. The fit is extended to the point where it intersects zero, which is recorded as the edge of the rarefaction wave. Results of this analysis can be found in Fig. 6 of the main manuscript.

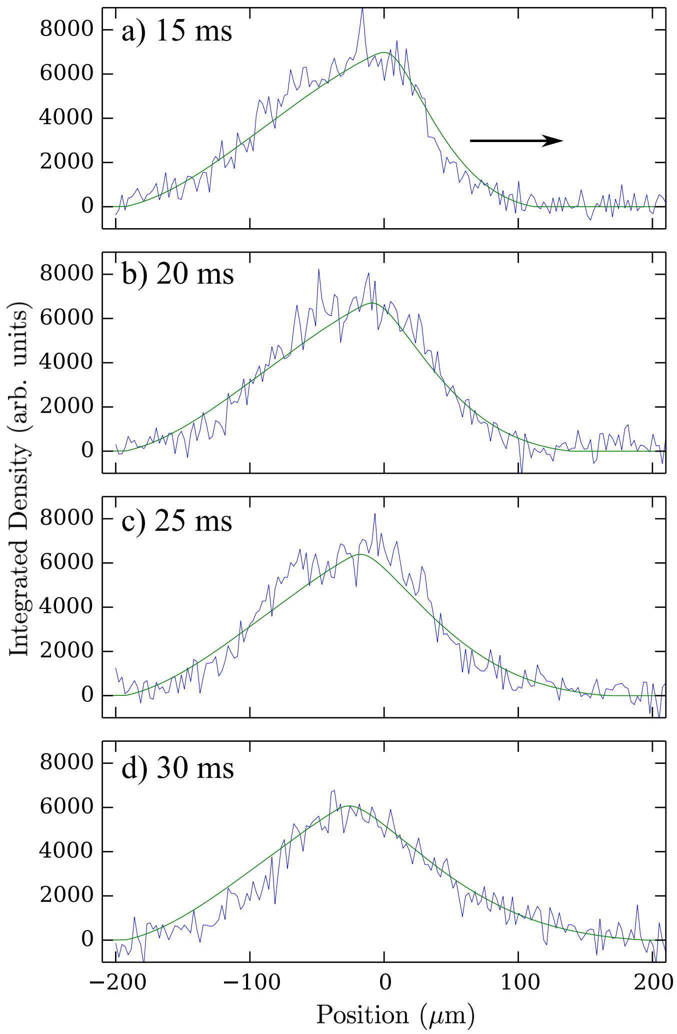

Supplementary Figure 5: *Integrated cross section of rarefaction waves.* Cross sections for experiment (blue) and numerics (green) are plotted for **a,** 15 ms, **b,** 20 ms, **c,** 25 ms, **d,** 30 ms, after the barrier has been jumped off.

SUPPLEMENTARY REFERENCES

[1] LeVeque, R. J. *Finite Volume Methods for Hyperbolic Problems.* Cambridge University Press (2002).

[2] Hoefer, M. A., Ablowitz, M. J. & Engels, P. Piston dispersive shock wave problem. *Phys. Rev. Lett.* **100,** 084504 (2008).

[3] Mateo, A. M. & Delgado, V. Effective mean-field equations for cigar-shaped and disk-shaped Bose-Einstein condensates. *Phys. Rev. A* **77,** 013617 (2008).

[4] Menotti, C. & Stringari, S. Collective oscillations of a one-dimensional trapped Bose-Einstein gas. *Phys. Rev. A* **66,** 043610 (2002).
